# Cardiovascular outcomes in type 1 and type 2 diabetes

**DOI:** 10.1007/s00125-022-05857-5

**Published:** 2023-01-14

**Authors:** Annika Rosengren, Pigi Dikaiou

**Affiliations:** 1 Department of Molecular and Clinical Medicine, Institute of Medicine, Sahlgrenska Academy, University of Gothenburg, Gothenburg, Sweden; 2 Region Västra Götaland, Department of Medicine, Geriatrics and Emergency Medicine, Sahlgrenska University Hospital, Östra Hospital, Gothenburg, Sweden; 3 Department of Endocrinology, Sahlgrenska University Hospital, Gothenburg, Sweden

**Keywords:** Cardiovascular disease, Glycaemic control, Review, Risk factors, Type 1 diabetes, Type 2 diabetes

## Abstract

Diabetes is one of the most prevalent cardiometabolic disorders on the planet. Type 1 diabetes accounts for only a minority of all cases (recently estimated to be ~2% globally); however, since this is a disorder with an early onset, many people live with type 1 diabetes for a long time. CVD and premature death are the main long-term outcomes for both types of diabetes; however, the type of diabetes that carries the highest risk of these outcomes is a controversial topic and has not been widely studied. Because of the association between diabetes and CVD, the rise in type 2 diabetes prevalence over the past decades has huge effects on global health. The excess risk in people with diabetes compared with those without depends, to a large extent, on the presence of other factors, such as general cardiovascular risk factors (e.g. elevated LDL-cholesterol, hypertension and smoking) and also factors that are more specific to diabetes (e.g. HbA1c, and micro- and macroalbuminuria). Some contributory factors are modifiable, while others are not, such as age, sex and type of diabetes. Older people with type 2 diabetes who have risk factors that are under control can achieve levels of CVD risk that are similar to that of the general population, while younger individuals with type 1 diabetes are mostly unable to achieve similar levels of risk, probably because of long and cumulative exposure to raised blood glucose levels. Despite reports of declining rates of CVD among people with type 1 and type 2 diabetes, rising rates of both types of diabetes lead to a continuing rise in the number of people with cardiometabolic disorders worldwide, offsetting the progress made in many countries. Comparison between individuals with type 1 and type 2 diabetes with respect to risk of CVD is fraught with difficulties and highly dependent on other, concomitant factors, some of which are modifiable and others not. Nonetheless, as a whole, what matters most in determining the management of diabetes is absolute risk and lifetime risk. Life-long efforts to achieve glycaemic control, control of lipids and hypertension, and not smoking are key to prevention, with a healthy lifestyle and pharmacological therapy to be implemented as needed.

**Graphical abstract:**

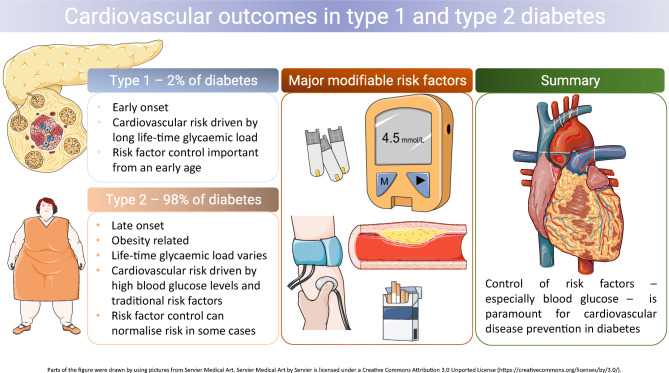

**Supplementary Information:**

The online version contains a slideset of the figures for download, which is available to authorised users at 10.1007/s00125-022-05857-5.

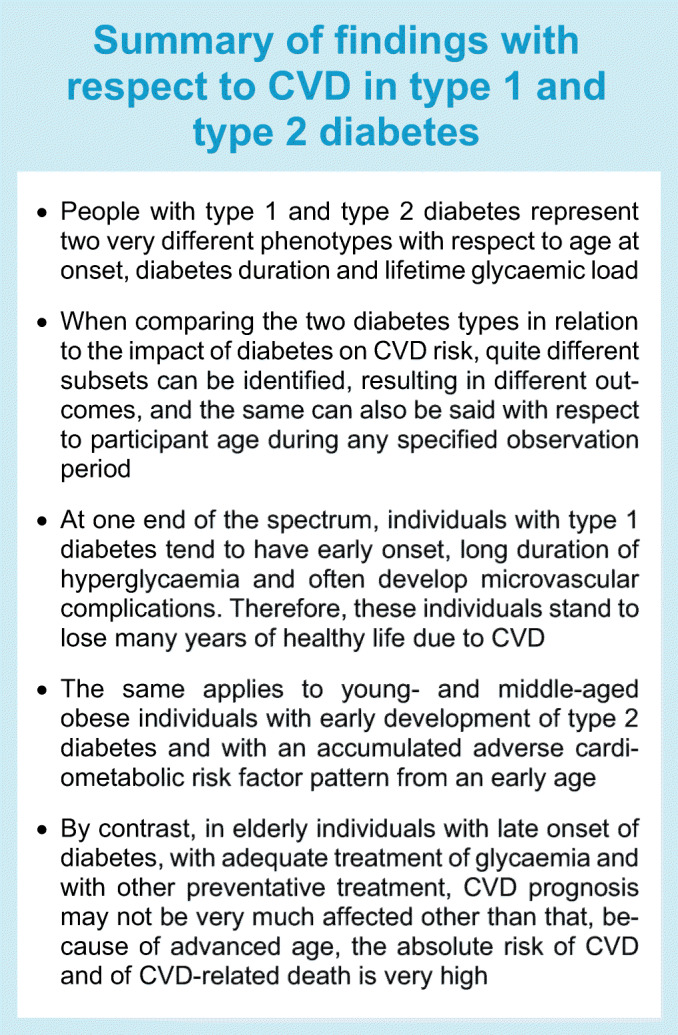



## Epidemiology of CVD in type 1 and type 2 diabetes

The global burden of diabetes is increasing [1], mainly in response to changes in human behaviour and lifestyle [2]. Diabetes is one of the most prevalent cardiometabolic disorders on the planet, estimated to have affected 10.5% of adults aged 20–79 years in 2021 [3]. Type 2 diabetes, representing the majority of all cases of diabetes, is a major public health challenge. The number of individuals with diabetes is currently estimated to rise from 536.6 million to 783.2 million by 2045. Because of recent developments with respect to the COVID-19 pandemic, war in Europe and climate change, which all threaten food supply and transportation [4], future predictions with regard to population body weight, obesity and incident type 2 diabetes are, however, more uncertain.

The largest increases in type 2 diabetes have been demonstrated in low- and middle-income countries, where rates now, in many instances, surpass those in high-income countries [5]. This is owing to changes in physical activity and diet, resulting in what is sometimes expressed as an ‘obesogenic environment’ [6]. Increasing numbers of young people with diabetes worldwide means that more individuals will live longer with diabetes. Given the high risk of CVD in diabetes and that diabetes management is often suboptimal [7], many individuals will experience early cardiovascular complications, lose earning power and struggle to provide for their families.

Previously, it was estimated that, of all individuals with diabetes, the proportion with type 1 diabetes was within the range of 5–15%; this was mostly based on data from high-income countries [2, 8, 9]. However, including data from low- and middle-income countries in calculations results in a substantially reduced proportion of individuals with type 1 diabetes, which was recently estimated to be only about 2% [10].

Even though type 1 diabetes is among the most common chronic diseases in children [11], many adults live with type 1 diabetes, either after onset in childhood or adolescence, or because of later onset. This means that a large part of the population with type 1 diabetes are adults at an age where CVD is a substantial risk. Conversely, with childhood obesity rates rising, type 2 diabetes is now a growing part of paediatric diabetes [12]. Accordingly, more children and adolescents with diabetes will be at increased risk of developing cardiovascular risk factors as young adults [13].

Type 1 diabetes incidence is also increasing [14–16], but not in a uniform manner. Recent data suggest a marked variation across countries and regions worldwide, potentially in response to heterogeneity in external factors [17]. Among factors postulated to be of interest are perinatal factors [18], and some hygienic factors that might indicate lack of microbial exposure in early life [19], while findings with respect to body weight and incident type 1 diabetes have been divergent [20, 21]. Data obtained from the Global Burden of Disease study described temporal trends in the incidence of different types of diabetes mellitus from 1990 to 2017 at global, regional and national levels [22], with a higher increase in estimated annual percentage change in type 2 diabetes compared with type 1 diabetes. Some studies have found an increase in type 1 diabetes in youth but not in adults [16, 23]. The reason for the increase in type 1 diabetes incidence has, however, been less systematically studied as compared with type 2 diabetes.

**Fig. 1:**
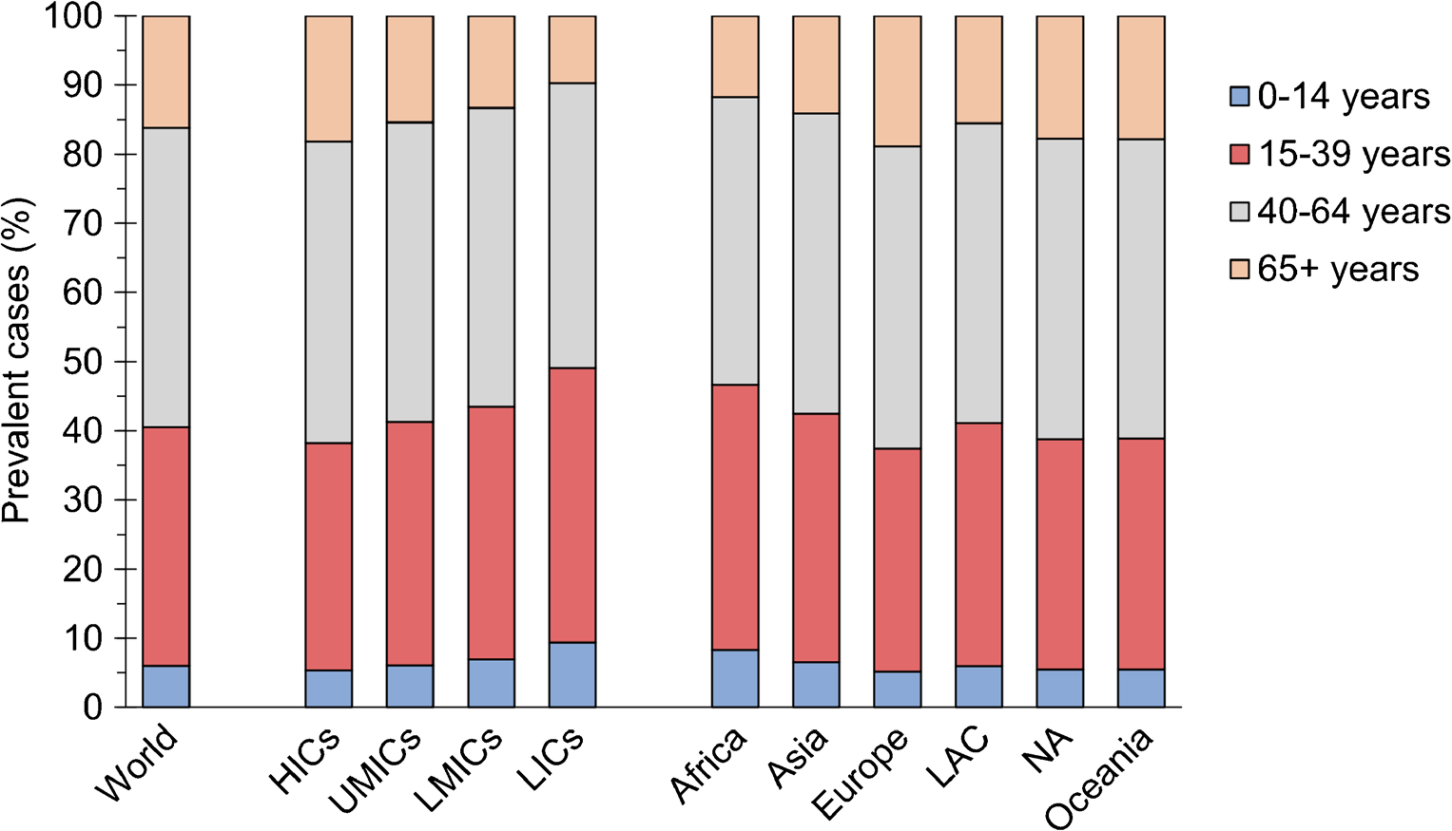
Distribution of prevalent cases of type 1 diabetes by current age, and by income groups and regions defined by UN population estimates. HICs, high-income countries; LAC, Latin America and the Caribbean; LICs, lower income countries; LMICs, lower middle-income countries; NA, North America; UMICs, upper middle-income countries. Reproduced from [10] under the terms of the Creative Commons Attribution 4.0 International License (http://creativecommons.org/licenses/by/4.0/), which permits unrestricted use, distribution, and reproduction in any medium. This figure is available as part of a downloadable slideset

In a recent global study, about 60% of all people with type 1 diabetes (estimated to be at 9 million in 2017) were above 40 years of age, representing an age where adverse cardiovascular-risk-factor patterns are highly prevalent and cardiovascular complications start to be clinically apparent (Fig. 1). Although only 17% of the world’s population reside in high-income countries, 49% of all new cases of type 1 diabetes occurred in these countries [10].

## Definitions and pathophysiology of different types of diabetes

Diabetes is classified into two main forms, type 1 and type 2 diabetes, but it is becoming increasingly clear that both types of diabetes are heterogeneous, in particular type 2 diabetes [24–26]. Type 1 diabetes is primarily the result of immune-mediated destruction of beta cells, while type 2 diabetes represents a broader spectrum of beta cell dysfunction coupled with insulin resistance. The net result, irrespective of type of diabetes, is hyperglycaemia, and when this occurs, people with all forms of diabetes are at risk for developing the same complications [27]. Still, rates of progression differ according to chronological age, age at onset [28, 29], HbA_1c_ levels [30–33], socioeconomic status [34] and other factors, as specified below.

Although mean age at onset in type 1 and type 2 diabetes differs by decades, both types can occur at any age [35]. Type 1 diabetes also includes latent autoimmune diabetes in adults (LADA), described as an intermediate of type 1 and type 2 diabetes, showing a faster progression to insulin therapy than that seen with type 2 patients. LADA is also associated with overweight/obesity in support of the hypothesis that, even in the presence of autoimmunity, excessive weight with resultant insulin resistance could promote onset of diabetes [26].

Late-life onset of type 1 diabetes is sometimes misdiagnosed as type 2, particularly since type 1 diabetes onset among older adults is often not as abrupt as in adolescents and younger adults [36]. Conversely, onset of type 1 diabetes in younger people can sometimes be mistaken for type 2 diabetes because they have overweight/obesity upon diagnosis. The distribution of BMI among children and adults with type 1 diabetes is similar to that of the general population [37], and therefore a proportion of individuals with early onset type 1 diabetes will be overweight or obese, although not to the same extent as individuals with type 2 diabetes.

## Risk factors for CVD in diabetes

Cardiovascular disorders have repeatedly been shown to be more common among people with type 1 and type 2 diabetes, as compared with people without diabetes. Cardiovascular disorders comprise CHD, including acute myocardial infarction (AMI) [38, 39], several types of stroke [40], heart failure [31, 32] and peripheral artery disease [41].

The excess risk of CVD in those with diabetes compared with people without depends, to a large extent, on the presence or absence of other factors. Factors that apply to all, irrespective of diabetes status are, for example, elevated LDL-cholesterol, hypertension and smoking. Other factors are more specific to diabetes, such as HbA_1c_ levels and micro- and macroalbuminuria. Background factors that are not modifiable are age, sex and type of diabetes. Older people with diabetes have higher absolute risk for CVD as compared with younger people with diabetes, while relative risk is lower when comparing individuals with and without diabetes of the same sex and age. Women with type 1 diabetes have about a 40% greater excess risk of mortality from any cause, and twice the excess risk of vascular events, compared with men with type 1 diabetes [42]. Type 2 diabetes confers a stronger excess risk of CVD in women than in men, with women being reported to have a 27% higher relative risk of stroke and a 44% higher relative risk of CHD compared with men [43]. Accordingly, women with diabetes of either type lose a substantial part of the normal female protection against CVD, in particular CHD [44].

In type 1 diabetes, optimal levels of modifiable risk factors are associated with lower risk of CVD. A cohort study based on the Swedish National Diabetes Registry followed 33,333 individuals with type 1 diabetes and 166,529 population control individuals without diabetes, matched for age, sex and county for a mean of 10.4 years [45]. The multiple-adjusted HR for AMI in individuals with type 1 diabetes with five predefined risk factors (HbA_1c_, BP, LDL-cholesterol, micro- or macroalbuminuria, and smoking) at target was 1.8, while having none of the five risk factors at target was associated with an adjusted HR of 12.3 as compared with the control group. A similar association was found for heart failure hospitalisation. Even with all risk factors at target, excess risk for heart failure remained significantly higher, by 97%, in comparison with the control group.

The importance of keeping blood glucose levels under control in type 1 diabetes is supported by intervention studies [46]. The DCCT compared individuals with type 1 diabetes randomly assigned to intensive vs conventional therapy for a mean of 6.5 years. During 30 years of follow-up, those assigned to intensive therapy had a reduced incidence of any CVD and of major cardiovascular events (non-fatal myocardial infarction, stroke or cardiovascular death) by about a third compared with the control group [47].

Even so, predisposition to CVD in type 1 diabetes is only partly attributable to traditional risk factors, with cardiovascular risk scores being found to work for the general population and those with type 2 diabetes but being poorly applicable to those with type 1 diabetes [48]. Signs of arterial dysfunction, such as arterial stiffness, might be one type of contributing factor. For example, pulse pressure, which reflects arterial stiffness, has been reported to increase at a younger age in type 1 diabetes as compared with healthy control individuals [49]. A recent study found arterial stiffness in the small resistance arteries to be independently associated with outcomes such as all-cause mortality and a composite of cardiovascular and/or diabetes-related mortality in type 1 diabetes [50]. Reduced coronary flow reserve, indicating early impairment of coronary vascular reactivity, has been shown in young individuals with type 1 diabetes, compared with healthy matched volunteers [51].

With respect to type 2 diabetes, individuals with this condition are phenotypically similar to patients with CVD, and the relation is bidirectional, with patients with CVD being shown to be more likely to develop diabetes even when not being diabetic at onset [52]. In individuals with type 2 diabetes, another study based on data from the Swedish National Diabetes Registry showed that a HbA_1c_ level over the target range was the strongest predictor of stroke and AMI, while smoking was the strongest predictor of death [38]. The risk of cardiovascular outcomes in individuals with diabetes with all risk factors within predefined target ranges was not higher than in control individuals without diabetes, except for heart failure (HR 1.45 [95% CI 1.34, 1.57]).

Similar to type 1 diabetes, there is support from intervention studies in type 2 diabetes for the importance of intensive glycaemic control, as summarised in current recommendations and guidelines [53], although less stringent control may be applicable in elderly individuals with type 2 diabetes. In a meta-analysis [54] that used data from five randomised controlled trials (UK Prospective Diabetes Study [UKPDS], PROspective pioglitAzone Clinical Trial In macroVascular Events [ProActive], Action in Diabetes and Vascular Disease: PreterAx and DiamicroN Modified Release Controlled Evaluation [ADVANCE], Veterans Affairs Diabetes Trial [VADT] and Action to Control Cardiovascular Risk in Diabetes [ACCORD] [55–60]), it was found that intensive vs standard glycaemic control significantly reduced coronary events, but did not significantly affect all-cause mortality. Later analyses of some of these trials have demonstrated that the effect on CVD may be explained by glycaemic legacy effects, with historical HbA_1c_ values having a greater impact than recent HbA_1c_ values on myocardial infarction incidence [61], although this has not been universally found [62].

## Cardiovascular outcomes in type 1 and type 2 diabetes

**Fig. 2:**
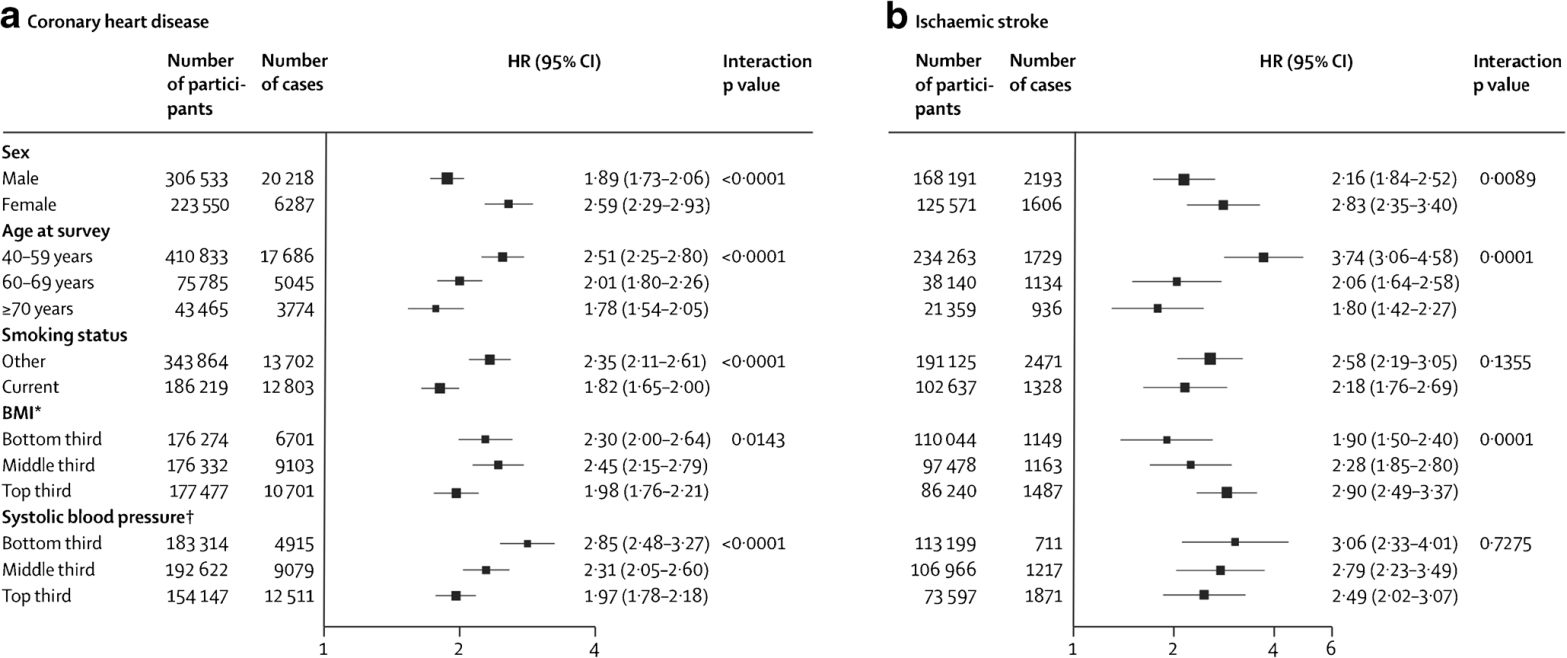
HRs for (**a**) CHD and (**b**) ischaemic stroke in people with diabetes at baseline vs those without, by individual characteristics. HRs were adjusted for age, smoking status, BMI and systolic BP, and, where appropriate, stratified by sex. *BMI categories: bottom third, <23.8 kg/m^2^ (mean: 21.7 kg/m^2^); middle third, 23.8–<27 kg/m^2^ (mean: 25.3 kg/m^2^); top third, ≥27 kg/m^2^ (mean: 30.7 kg/m^2^). ^†^Systolic BP categories: bottom third, <123 mmHg (mean: 113 mmHg); middle third, 123–<141 mmHg (mean: 132 mmHg); top third, ≥141 mmHg (mean: 157 mmHg). Reproduced from [63], © 2010 Elsevier Ltd, published as Open Access. This figure is available as part of a downloadable slideset

Most of the vast literature concerning diabetes and cardiovascular outcomes relates to type 2 diabetes. In a meta-analysis of individual records of 698,782 people without initial vascular disease from 102 prospective studies, adjusted HRs for diabetes vs no diabetes were 2.00 (95% CI 1.83, 2.19) for CHD, 2.27 (95% CI 1.95, 2.65) for ischaemic stroke, 1.56 (95% CI 1.19, 2.05) for haemorrhagic stroke, 1.84 (95% CI 1.59, 2.13) for unclassified stroke and 1.73 (95% CI 1.51, 1.98) for other vascular deaths, with little change after adjustment for other relevant factors [63]. HRs for CHD associated with diabetes vs no diabetes were higher in women than in men, in younger than in older participants, and in those with fatal vs non-fatal disease. Figure 2 shows HRs for CHD and ischaemic stroke in people with diabetes at baseline vs those without, by individual characteristics.

**Fig. 3:**
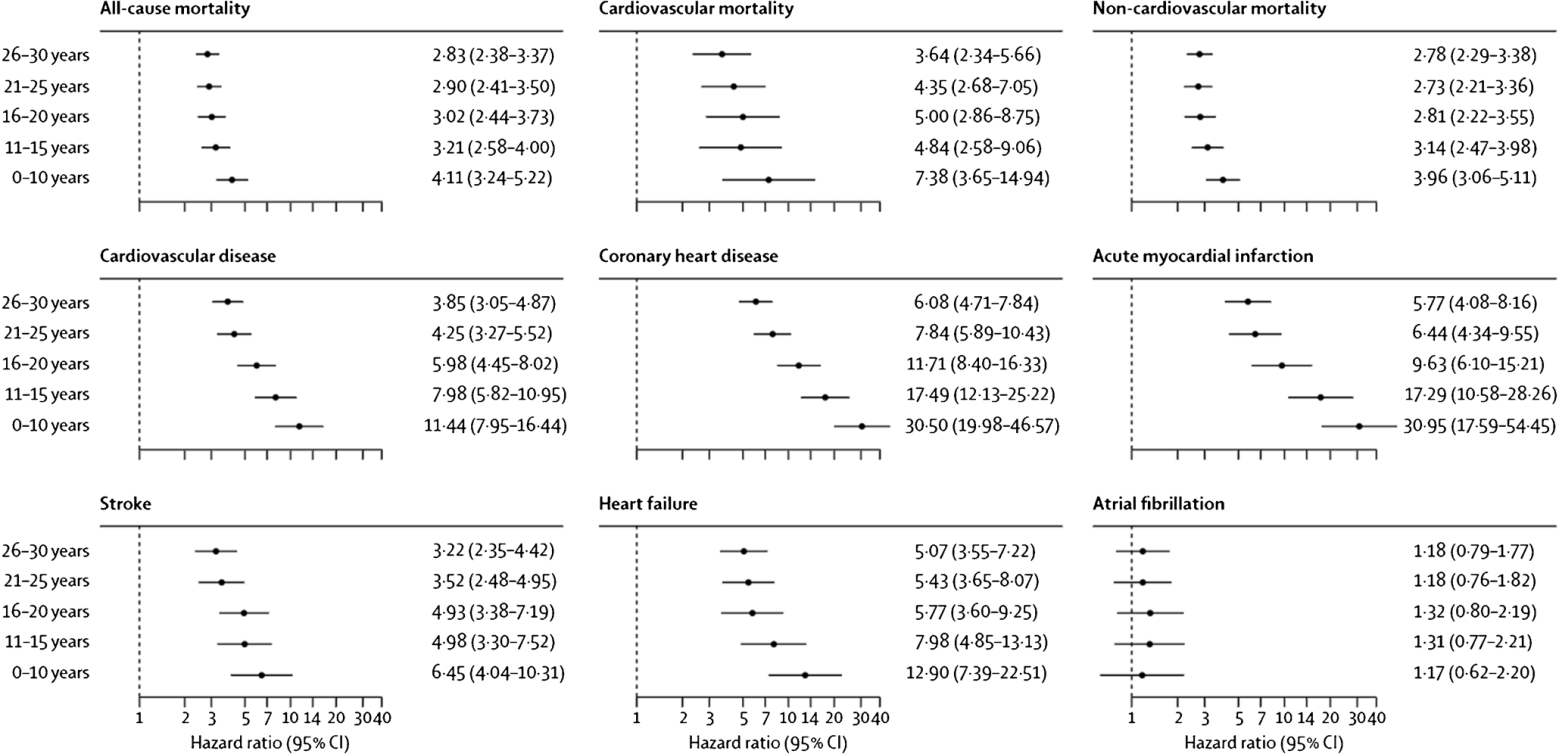
Forest plot showing adjusted HRs for mortality and cardiovascular outcomes, according to age at type 1 diabetes diagnosis. Analyses were based on Cox regression and adjusted for pre-existing comorbidities, calendar year, income, country of birth, marital status, educational attainment, and duration of diabetes. Matched controls served as a reference group for all models. Reproduced from [28], with permission from Elsevier. This figure is available as part of a downloadable slideset

In comparison, a meta-analysis of ten observational studies was conducted, involving 166,027 patients with type 1 diabetes and matched control individuals from the general population [64]. With respect to CHD, the overall relative risk was 9.38 (95% CI 5.56, 15.82), and for myocardial infarction it was 6.37 (95% CI 3.81, 10.66), compared with controls without diabetes. Notably, however, estimates for CHD were based on two studies only, and for AMI on three studies. A Swedish study based on the National Diabetes Register (NDR) was common to both outcomes [28], demonstrating extremely high relative risks in individuals with type 1 diabetes with onset at an age of 10 years and younger compared with matched control participants from the general population. These relative risks, however, were lower among those with onset after 20 years of age (Fig. 3), again likely to be reflecting the toxic effect of long-standing hyperglycaemia among people with diabetes onset during an era with fewer options available to obtain normoglycaemia.

Comparing risks of cardiovascular outcomes between people with type 1 and type 2 diabetes is difficult, not only because of the differences in phenotype but also because absolute risks vary substantially depending on many other factors. This applies to comparing relative risks in people with type 1 and type 2 diabetes, as well as excess risks compared with individuals without diabetes. For a start, unmodifiable risk factors, including mean age, age at onset and diabetes duration, are vastly different between the two types. Data from the NDR, which has high national coverage, provide descriptive data in representative and large groups of individuals with both types of diabetes. In one study, which did not primarily focus on comparing outcomes but included baseline data from 36,869 individuals with type 1 diabetes and 457,473 individuals with type 2 diabetes [65], the mean age at entry was 35.3 years for type 1 diabetes and 65.2 years for type 2 diabetes, a difference of 30 years. The mean duration of diabetes was substantially longer in type 1 diabetes (20.0 years) in comparison with those with type 2 diabetes (5.7 years). The mean HbA_1c_ level was 8.2% (66.0 mmol/mol) in individuals with type 1 diabetes and 7.1% (54.5 mmol/mol) in those with type 2 diabetes. Similarly, a study of nearly 1.2 million people with diabetes (6% with type 1 diabetes), registered on the Australian diabetes registry, compared trends in cause-specific mortality between 2010 and 2019. Median age at diagnosis for type 1 diabetes was 22.3 years and for type 2 diabetes, it was 58.2 years [66], with smaller differences in diabetes duration (17.6 years and 10.3 years, respectively). Differences in population characteristics illustrate that there are many inherent difficulties when comparing outcomes in type 1 and type 2 diabetes.

Among the general population, higher age is by far the most important determinant of absolute risk. Still, lower age at diabetes onset is an important determinant of survival, as well as of cardiovascular outcomes in both type 1 and type 2 diabetes [28, 29]. Individuals with type 1 diabetes, while on average being younger and with lower short-term absolute risk, will have had much longer exposure to dysglycaemia than those with type 2 diabetes. The latter, however, have higher absolute risk because they are older. Accordingly, when comparing macrovascular complications in people with type 1 and type 2 diabetes, these differences make for very unbalanced comparison groups, with baseline factors working in opposing directions.

## Comparison of cardiovascular outcomes by type of diabetes

Irrespective of type of diabetes, onset at a young age, as compared with an older age, indicates longer exposure to hyperglycaemia, with a higher risk of micro- and macrovascular complications [28, 29]. Even so, compared with type 1 diabetes, there is a much higher presence of cardiovascular risk factors in individuals with early onset type 2 diabetes. So far, relatively few studies exist that compare CVD outcomes in those with type 1 vs type 2 diabetes. Table 1 summarises findings from some relevant studies.

**Table 1:** Selected publications comparing CVD in type 1 and type 2 diabetes

Publication	Setting and participant selection	Population characteristics	Findings	Comment
Constantino et al [67]	Records from the Royal Prince Alfred Hospital Diabetes Clinical Database matched with the Australian National Death Index to establish mortality outcomes from 1986 until June 2011. Clinical and mortality outcomes in individuals with T2DM (*n*=354)*,* age of onset 15–30 years, were compared with those with T1DM, primarily with T1DM individuals (*n*=470) with a similar age of onset.	Age of diabetes onset: T2DM, 25.6 years; T1DM, 22.0 years. Duration of diabetes: T2DM, 11.6 years; T1DM, 14.7 years. Age at baseline: T2DM, 40.4 years; T1DM, 38.9 years.	After median observation period of >20 years, young-onset T2DM found to be more lethal diabetes phenotype and was associated with greater mortality, mostly driven by CVD mortality, more complications and unfavourable CVD risk factors vs T1DM.	
Eppens et al [68]	Clinic-based cross-sectional study of individuals with T1DM (*n*=1433) and T2DM (*n*=68), aged <18 years, from New South Wales, Australia.	Mean age: T1DM, 15.7 years; T2DM, 15.3 years. Mean diabetes duration: T1DM, 6.8 years; T2DM, 1.3 years. HbA_1c_: T1DM, 8.5% (69 mmol/mol); T2DM, 7.3% (56 mmol/mol).	Microalbuminuria and hypertension significantly more common in T2DM vs T1DM. Despite shorter diabetes duration, microalbuminuria found in >25% of T2DM vs 6% of T1DM.	Cross-sectional study, no prospective data.
Dabelea et al [69]	Observational study from 2002 to 2015 in 5 US locations, including participants with T1DM (*n*=1746) and T2DM (*n*=272) diagnosed at <20 years old.	Mean diabetes onset: T1DM, 10.0 years; T2DM, 14.2 years. Age: T1DM, 17.9 years; T2DM, 22.1 years. Major difference prevalence of obesity: T1DM, 14.3%; T2DM, 72.1%.	Prevalence of each outcome estimated at age 21 years by diabetes type. After adjustment for established risk factors, T2DM group had significantly higher odds of DKD, retinopathy and peripheral neuropathy vs T1DM, but no significant difference in odds of arterial stiffness, hypertension, or autonomic neuropathy.	No CV outcomes.
Hockett et al [72]	A US and an Indian registry dataset with demographic and clinical data were harmonised. Key characteristics from youth with T1DM and T2DM, aged <20 years and newly diagnosed between 2006 and 2010 were compared.	There were 1899 US youth with T1DM and 384 with T2DM who completed a baseline research visit. There were 2104 Indian youth with T1DM and 227 with T2DM who completed a baseline visit.	US vs. Indian patients were diagnosed at younger ages for T1DM and T2DM (10.1 vs 10.5 years, *p*<0.001 and 14.7 vs 16.1 years, *p*<0.001, respectively). For T2DM, the US database had a higher proportion of people with low SES than in India. For T1DM and T2DM, US youth had a higher BMI, lower BP, and lower HbA_1c_ than Indian youth.	
Luk et al [73]	*N*=2323 Chinese individuals (T1DM, *n*=209; normal-weight T2DM, *n*=636; overweight T2DM, *n*=1478) from the Hong Kong Diabetes Registry underwent detailed clinical assessment during 1995–2004.	Mean age: T1DM, 27.8 years; normal-weight T2DM, 41.9 years; overweight T2DM, 40.8 years. Time since diabetes diagnosis: T1DM, 8 years; normal-weight T2DM, 7 years; overweight T2DM, 5 years.	Over median follow-up of 9.3 years, overweight T2DM had highest incidence of CVD, followed by normal-weight T2DM group. Compared with T1DM, overweight T2DM group had greater hazard of progression to CVD (HR 15.3 [95% CI 2.1, 112.4]) following adjustment for age, sex and disease duration. The association became nonsignificant upon additional adjustment for CVD risk factors.	The number of events was limited, particularly in the T1DM cohort. CIs for the estimates were very wide, precluding firm conclusions.
Juutilainen et al [75]	Cohort study of individuals with T1DM (*n*=173) and T2DM (*n*=834), aged 45–64 years at baseline and free of CVD, identified from drug reimbursement registry in Finland. Age of diabetes onset was >30 years in both diabetes groups. Nondiabetic participants (*n*=1294) from a random sample were invited for comparison.	Population baseline characteristics not tabulated. Compared with T2DM, T1DM group stated to be younger, leaner, with lower prevalence of hypertension, lower BP, higher HDL-c, lower TG, longer diabetes duration and lower estimated creatinine clearance.	After 18 years follow-up, impact of T1DM and T2DM on CVD-related mortality was similar. Effect of increasing hyperglycaemia on risk of CVD mortality more pronounced in T1DM vs T2DM.	Compared only maturity-onset diabetes.
Allemann et al [76]	Cohort study of T1DM (*n*=225) and T2DM (*n*=308) participants recruited from 231 Swiss local practitioners. Participants followed for 30 years.	Mean age at baseline: T1DM, 43.0 years; T2DM, 46.8 years. Mean diabetes duration: T1DM, 15.5 years; T2DM, 9.2 years.	Compared with general Swiss population, T1DM and T2DM groups had increased risk of CVD mortality (SMR 5.6 [95% CI 4.8, 6.6]) but SMRs did not significantly differ between T1DM and T2DM.	Not clear how patients were selected.
Amutha et al [77]	Individuals with T1DM (*n*=108) and T2DM (*n*=90) were recruited from a tertiary diabetes centre in Chennai, India. Participants were diagnosed at 10–25 years old and did not have any evidence of diabetes complications at diagnosis.	Mean age at diagnosis: T1DM, 17.1±4.2 years; T2DM 21.6±3.6 years.	In Cox regression analysis, after adjustment for age, HbA_1c_, BP and serum cholesterol, T2DM group had 2.11 times (95% CI 1.27, 3.51) higher risk of developing any diabetes complication vs T1DM, indicating that young-onset T2DM has a more aggressive disease course than T1DM.	Not powered for separate CV outcomes.
Kiss et al [78]	Young adults with T1DM recorded in the Hungarian National Health Insurance Fund between 2001 and 2014 (*n*=11,863) and a similar age T2DM population (*n*=47,931).	Mean age: T1DM, 21.6 years; T2DM, 33.5 years. Mean follow-up: T1DM, 6.5 years; T2DM, 6.6 years.	HRs (T1DM vs T2DM): all-cause mortality, 2.17 (95% CI 1.95, 2.41); MI, 0.90 (95% CI 0.70, 1.13); stroke, 1.06 (95% CI 0.87, 1.29).	No data on diabetes duration or glycaemic control.
Lee et al [79]	Korean adults with T1DM (*n*=9397) vs those without diabetes (*n*=18.5 million) or with T2DM (*n*=1.9 million), using Korean National Health Insurance Service datasets. T1DM accounted for 0.5% of all diabetes.	Mean age: no diabetes, 45.9 years; T1DM, 56.2 years; T2DM, 57.8 years. Mean follow-up: 4.6 years. Mean BMI: T1DM, 24.1 kg/m^2^; T2DM, 25.1 kg/m^2^.	Fully adjusted HRs (95% CIs) for incident MI (1.68 [1.49, 1.89]), hospitalised HF (2.11 [1.90, 2.33]), AF (1.61 [1.41, 1.83]) and all-cause death (1.88 [1.76, 2.01]) within mean follow-up of 4.6 years higher in T1DM vs T2DM.	Diabetes duration was dichotomously categorised as <5 years and ≥5 years because complete determination of diabetes duration was not feasible for those diagnosed >5 years before the baseline since past data were unavailable.

AF, atrial fibrillation; CV, cardiovascular; DKD, diabetic kidney disease; HDL-c, HDL-cholesterol; HF, heart failure; MI, myocardial infarction; SES, socioeconomic status; SMR, standardised mortality ratio; T1DM, type 1 diabetes; T2DM, type 2 diabetes; TG, triglycerides

**Fig. 4:**
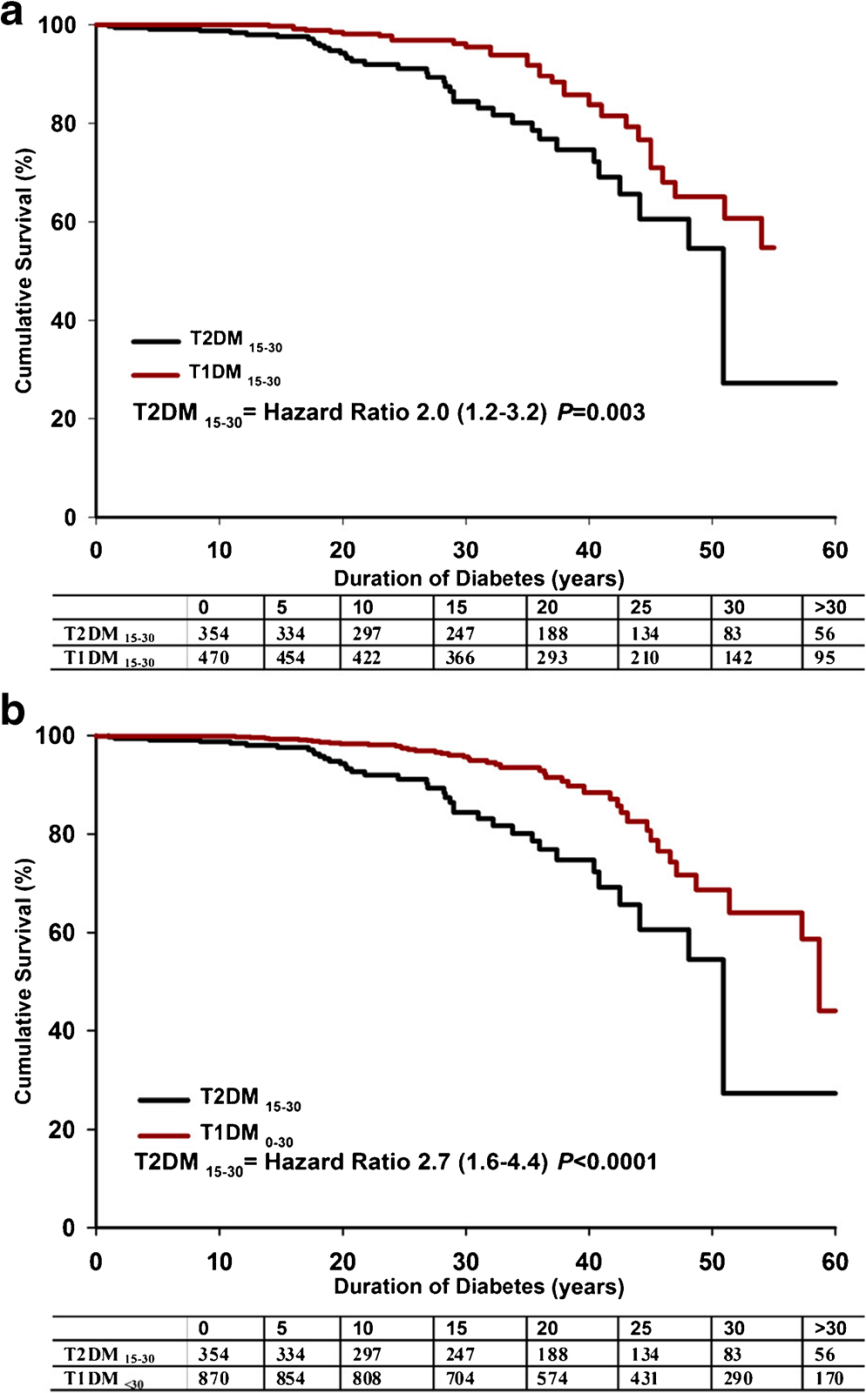
(**a**) Kaplan–Meier survival curve for individuals with type 2 diabetes (T2DM), aged 15–30 years (*n*=354), and type 1 diabetes (T1DM), aged 15–30 years (*n*=470). (**b**) Kaplan–Meier survival curve for individuals with type 2 diabetes, aged 15–30 years (*n*=354) and all individuals with type 1 diabetes (age of onset <30 years; *n*=870). Reprinted from [67] under the terms of the Creative Commons Attribution-NonCommercial-NoDerivs 3.0 Unported License (https://creativecommons.org/licenses/by-nc-nd/3.0/), which permits copying and distribution in any medium or format in the unadapted form, for noncommercial purposes only. This figure is available as part of a downloadable slideset

In an Australian study, a clinical database was used to examine the long-term complications, between 1986 and 2011, in individuals aged 15–30 years at diabetes diagnosis [67]. Altogether, 470 individuals with type 1 diabetes and 354 individuals with type 2 diabetes were identified, with a mean diabetes duration of 14.7 and 11.6 years, respectively. Participants with type 1 and type 2 diabetes were followed from the age of 39 and 40 years, respectively. Mean BMI was 25.6 kg/m^2^ for those with type 1 diabetes and 32.2 kg/m^2^ for those with type 2 diabetes. Despite a shorter duration of diabetes and similar metabolic control, the type 2 diabetes cohort had more complications, with more albuminuria and abnormal biothesiometer findings, and a marked excess of prevalent macrovascular disease compared with those with type 1 diabetes (ischaemic heart disease: 12.6% vs 2.5%; stroke: 4.3% vs 0.7%). During a median observation period of over 20 years, when compared with those with type 1 diabetes, a higher proportion of patients with type 2 diabetes died (11% vs 6.8%; HR 2.0 [95% CI 1.2, 3.2]), after a significantly shorter disease duration and at a relatively early age. Figure 4 shows Kaplan–Meier survival curves for type 1 and 2 diabetes. Altogether, the authors concluded that type 2 diabetes with early onset was the more lethal of the two types of diabetes. Similarly, in a clinic-based cross-sectional study of 1433 participants with type 1 diabetes and 68 participants with type 2 diabetes aged <18 years from New South Wales, Australia, adolescents with type 2 diabetes had significantly higher rates of microalbuminuria and hypertension than those with type 1 diabetes, despite shorter diabetes duration and better glycaemic control [68], indicating a potentially higher risk for CVD with time.

In the US-based SEARCH for Diabetes in Youth registry study, children and adolescents diagnosed with diabetes at <20 years of age were identified from a population-based incidence registry network [69]. Of these, 1746 had type 1 diabetes, while 272 had type 2 diabetes (mean age 17.9 and 22.1 years, respectively), with a major difference in the prevalence of obesity (14.3% vs 72.1%, respectively). At age 21 years, and after nearly 8 years mean diabetes duration, the participants in the study underwent a range of examinations with respect to potential complications, showing overall higher age-adjusted prevalence of diabetic kidney disease in type 2 diabetes vs type 1 diabetes (19.9% vs 5.8%), retinopathy (9.1% vs 5.6%) and peripheral neuropathy (17.7% vs 8.5%), but not of cardiovascular autonomic neuropathy. Prevalence of arterial stiffness (47.4% vs 11.6%) and hypertension (21.6% vs 10.1%), both relevant for later development of cardiovascular complications, were also more common in type 2 diabetes vs type 1 diabetes. However, with the inclusion of multiple risk factors, including waist–height ratio, in the final analytical model, the associations of diabetes type with arterial stiffness and hypertension were no longer significant, indicating that differences in obesity (in particular central obesity) contributed to the differences observed between those with type 2 and type 1 diabetes. These findings support the view that young individuals with type 2 diabetes may be at higher risk of cardiovascular complications as compared with individuals with type 1 diabetes.

Young-onset type 2 diabetes was recently reviewed and termed as one of the most serious health challenges of the 21st century, which has seen the greatest relative increases in type 2 diabetes incidence and prevalence in younger adults (<40 years old) [70]. A more aggressive phenotype has been postulated in individuals diagnosed at an earlier vs later age, with a more rapid deterioration in beta cell function [71]. Again, most data are derived from western, high-income countries. However, in a study comparing individuals from the USA and India, with early onset type 1 and type 2 diabetes, aged <20 years and newly diagnosed between 2006 and 2010, there were some notable differences between diabetes types [72]. One of the main differences was age of onset of type 2 diabetes, which was 14.7 years in the USA and 16.1 years in India. In addition, US participants with young-onset type 2 diabetes were more often female and had lower socioeconomic status. Moreover, US participants with either type 1 or type 2 diabetes had higher BMI than their Indian peers. For example, in the type 2 diabetes cohort, 79% of US participants were obese (defined by BMI *z*-scores), as compared with 37% of Indian participants.

Further, the Hong Kong Diabetes Registry [73], established in 1995, consecutively enrolled individuals with type 1 and type 2 diabetes who were referred to the hospital for assessment. Of 2323 individuals with diabetes onset before the age of 40 years, 209 (9.0%) had type 1 diabetes, 636 (27.4%) had type 2 diabetes and were of normal weight, and 1478 (63.6%) had type 2 diabetes and were overweight (BMI ≥23 kg/m^2^ in this Asian population) [74]. Individuals with type 2 diabetes were older (normal-weight type 2 diabetes: 42 years old; overweight type 2 diabetes: 41 years old; type 1 diabetes: 28 years old), while those with type 1 diabetes had longer duration of diabetes. Overweight participants with type 2 diabetes had the highest prevalence of cardiometabolic risk factors. Over a median follow-up duration of 9.3 years, CVD incidence was highest in the overweight type 2 diabetes group and lowest in the type 1 diabetes group, with respective rates of 9.6, 5.1 and 0.6 events per 1000 person-years in the overweight type 2 diabetes, normal-weight type 2 diabetes and type 1 diabetes groups, respectively (*p*<0.01 for all comparisons). Overweight individuals with type 2 diabetes were 15 times more likely to develop a cardiovascular event, compared with individuals with type 1 diabetes, while adjustment for CVD risk factors annihilated the excess risk. There was no statistical difference between the normal-weight type 2 diabetes and type 1 diabetes groups. It must be noted, however, that statistical power was limited for the comparisons between the overweight and normal-weight type 2 diabetes groups with the type 1 diabetes group.

Few studies exist comparing middle-aged individuals with type 1 and type 2 diabetes with respect to long-term outcomes. A Finnish study comprised 173 participants with type 1 diabetes, 834 participants with type 2 diabetes and 1294 nondiabetic participants, aged 45–64 years at baseline and free of CVD [75]. The age of onset of diabetes was 30 years or older in both diabetes groups. At the 18 year follow-up, the impact of diabetes on cardiovascular mortality was similar between the type 1 and type 2 diabetes groups.

In a Swiss study that compared 225 individuals with type 1 diabetes and 308 individuals with type 2 diabetes aged 43 years and 47 years, respectively, with a mean diabetes duration of 15.5 years and 9.2 years, respectively, individuals were followed for 30 years [76]. There were 169 cardiovascular deaths, equating to 13.0 per 1000 person-years in the type 1 diabetes group and 17.8 per 1000 person-years in the type 2 diabetes group. Even more limited data exist that compare type 2 and type 1 diabetes in non-western populations; in a small study from Chennai, India, which investigated complications in type 1 and type 2 diabetes, those with type 2 diabetes had a doubled risk of developing any diabetes complication. However, the sample size was too small to yield any meaningful information on macrovascular complications for comparison between the two types of diabetes [77].

Two other recent large studies have come up with slightly conflicting results; in a retrospective cohort study, Kiss et al [78] identified all young adults (<40 years of age) with type 1 diabetes who were recorded in the database of the Hungarian National Health Insurance Fund between 2001 and 2014 (*n*=11,863) and compared them with a type 2 diabetes population of similar age (*n*=47,931). After adjustments, those with type 1 diabetes had approximately twice the risk of dying compared with those with type 2 diabetes, but there was no difference with respect to risk of myocardial infarction. By contrast, data from the Korean National Health Insurance Service datasets of preventive health check-ups from 2009 to 2016 were used to study CVD and mortality in individuals who were ≥20 years old, without baseline CVD (*N*=20,423,051) [79]. In fully adjusted models, rates of incident myocardial infarction, hospitalised heart failure, atrial fibrillation and all-cause death within a mean follow-up of 4.6 years, were significantly higher in the type 1 diabetes group than the type 2 diabetes group. Of note, mean age at baseline for type 1 and type 2 diabetes was 56 years and 58 years, respectively, while mean BMI was low, at 24 kg/m^2^ and 25 kg/m^2^, respectively, and there were no exact data on diabetes duration.

## Trends in outcomes in type 1 and type 2 diabetes

CVD occurrence in the population is a dynamic topic, with a marked decline in coronary mortality in western, high-income countries over the last decades [80]. In the general population, positive developments have been seen in many parts of the world with respect to prevention and treatment of CVD [81]. Still, this does not apply to obesity rates, which are increasing globally, or to CVD rates in low- and middle-income countries, which are also increasing. In contrast, cancer now surpasses CVD as a main cause of death in high-income countries [82]. In the USA, increasing numbers of cardiometabolic deaths (owing to heart disease, cerebrovascular disease and diabetes) in adults <65 years of age have been demonstrated and these have been linked to the recently observed decline in life expectancy [83].

In a study using interviewer-collected household survey data on the health status and behaviours of the US non-institutionalised population, major CVD-associated mortality was found to have declined from the late 1980s to 2015 in adults with diabetes, especially in men [84]. Large reductions were observed for mortality from ischaemic heart disease and stroke, although trends in heart failure- and arrhythmia-related deaths did not change. Notably, significant differences persisted across race/ethnicity and education groups. Ten year percentage change was greater in the group with (−32.7%) than without (−26.2%) diabetes. There was a lack of improvement among young adults, irrespective of diabetes status. Another analysis using data derived from the same study population concluded that virtually all of the decline in death rate among adults with diabetes was caused by the reduction in vascular disease-related deaths. Vascular disease accounted for almost half of deaths in people with diabetes in the early 1990s, falling to about one-third of deaths in 2010–2015 [85]. Type of diabetes was not specified but given the greater preponderance of type 2 diabetes, findings likely apply mainly to this group. A similar study from Sweden, comparing individuals with diabetes with controls selected from the general population, matched for age and sex, with respect to non-fatal and fatal outcomes [65], over the period 1998–1999 to 2012–2013, found that individuals with type 1 diabetes had a 40% greater reduction in cardiovascular outcomes than the control group, while individuals with type 2 diabetes roughly had a 20% greater reduction than control participants. While reductions in fatal outcomes were similar in those with type 1 diabetes and control individuals, those with type 2 diabetes had smaller reductions in fatal outcomes as compared with the control group.

One study [86] analysed over one million Australians with diabetes (7.3% with type 1 diabetes) registered on the National Diabetes Service Scheme between 2000 and 2011. There was a decrease in cardiovascular deaths in both types of diabetes, but this was not consistently seen across age groups, with younger people (<40 years old) with type 1 diabetes seeing smaller improvements in CVD-related and all-cause mortality than those aged 40–70 years. Worryingly, those with type 2 diabetes and <40 years old experienced an increase in all-cause mortality and no decline in CVD-related death.

## Conclusions and directions for future studies

Despite declining rates of CVD among people with type 1 and type 2 diabetes, as well as in the general population, growing rates of both types of diabetes is leading to a continuing rise in the number of people with cardiometabolic disorders worldwide. This will offset the progress in many countries with respect to prevention and treatment of CVD and lead to potential stalling of increasing life expectancy. For type 2 diabetes, the link to rising rates of obesity is evident, and data from several sources indicate that type 2 diabetes with onset at an early age is particularly deleterious with respect to both micro- and macrovascular complications. For type 1 diabetes, the cause for the rise in incidence is less clear and probably multifactorial, but the large variation in incidence, with a predominance of cases in high-income countries, indicates that rising obesity rates may play a role.

As illustrated by the literature discussed above, comparison between individuals with type 1 and type 2 diabetes with respect to risk of CVD is fraught with difficulties and highly dependent on other concomitant factors, some of which are modifiable and others not (see Text box). Even so, because findings, so far, are highly dependent on context and are inconclusive, the continuing comparison of outcomes is still of professional and public interest, not least for individuals with diabetes. What will matter most in determining management in any individual with diabetes, however, is absolute risk and lifetime risk. Accordingly, glycaemic control, control of lipids and hypertension, and a healthy lifestyle (not smoking, appropriate diet and physical activity) are key to prevention in diabetes of both types, with pharmacological therapy as needed. The prevention of diabetes and of its complications will require an integrated, international approach. These efforts, in particular with respect to glycaemic control, should start early, including identification and treatment of CVD risk factors, as recommended in multiple guidelines [53, 87].

## Supplementary information

Slideset of figures (PPTX 630 kb)

## Abbreviations

AMI: Acute myocardial infarction
LADA: Latent autoimmune diabetes in adults
NDR: National Diabetes Register
