# Supplementary material for: Cardiovascular outcomes in type 1 and type 2 diabetes
Source: Diabetologia. 2023 Jan 14;66(3):425–37. doi: 10.1007/s00125-022-05857-5 (PMC9840171; doi:10.1007/s00125-022-05857-5)
Supplement: Supplementary file 1 — (PPTX 630 kb) [file 125_2022_5857_MOESM1_ESM.pptx]

## Slide 1
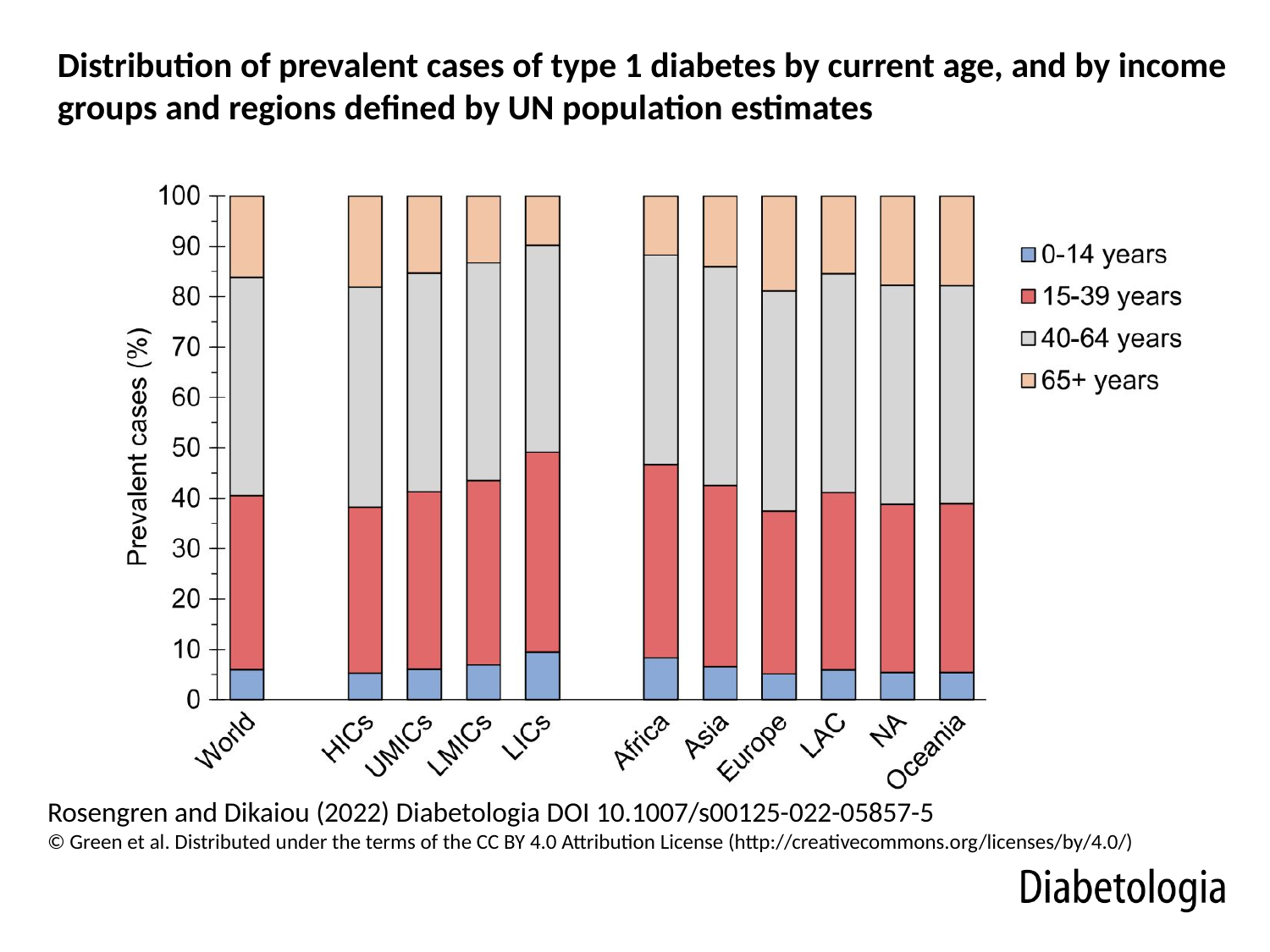

Distribution of prevalent cases of type 1 diabetes by current age, and by income groups and regions defined by UN population estimates
Rosengren and Dikaiou (2022) Diabetologia DOI 10.1007/s00125-022-05857-5
© Green et al. Distributed under the terms of the CC BY 4.0 Attribution License (http://creativecommons.org/licenses/by/4.0/)

## Slide 2
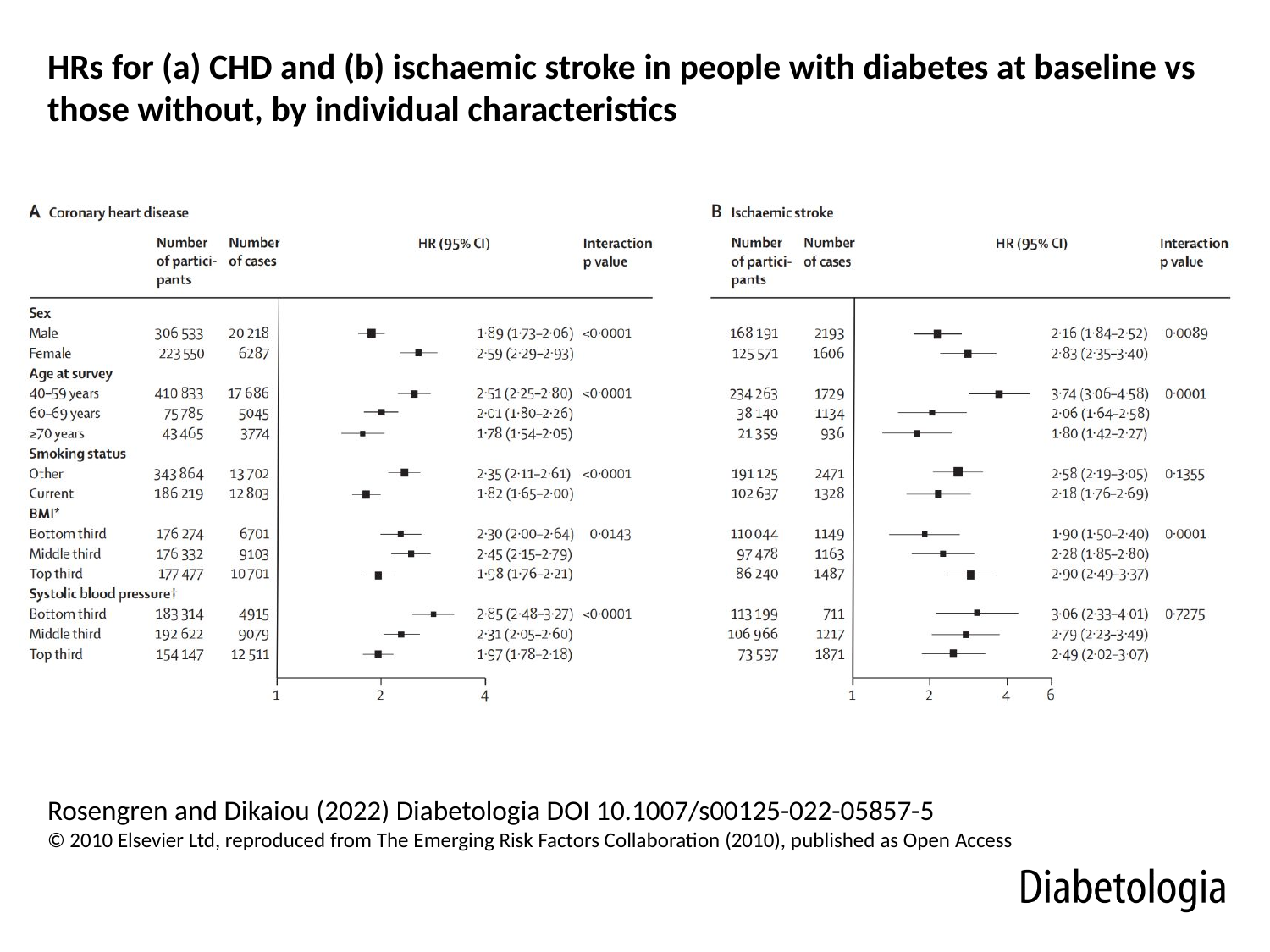

HRs for (a) CHD and (b) ischaemic stroke in people with diabetes at baseline vs those without, by individual characteristics
Rosengren and Dikaiou (2022) Diabetologia DOI 10.1007/s00125-022-05857-5
© 2010 Elsevier Ltd, reproduced from The Emerging Risk Factors Collaboration (2010), published as Open Access

## Slide 3
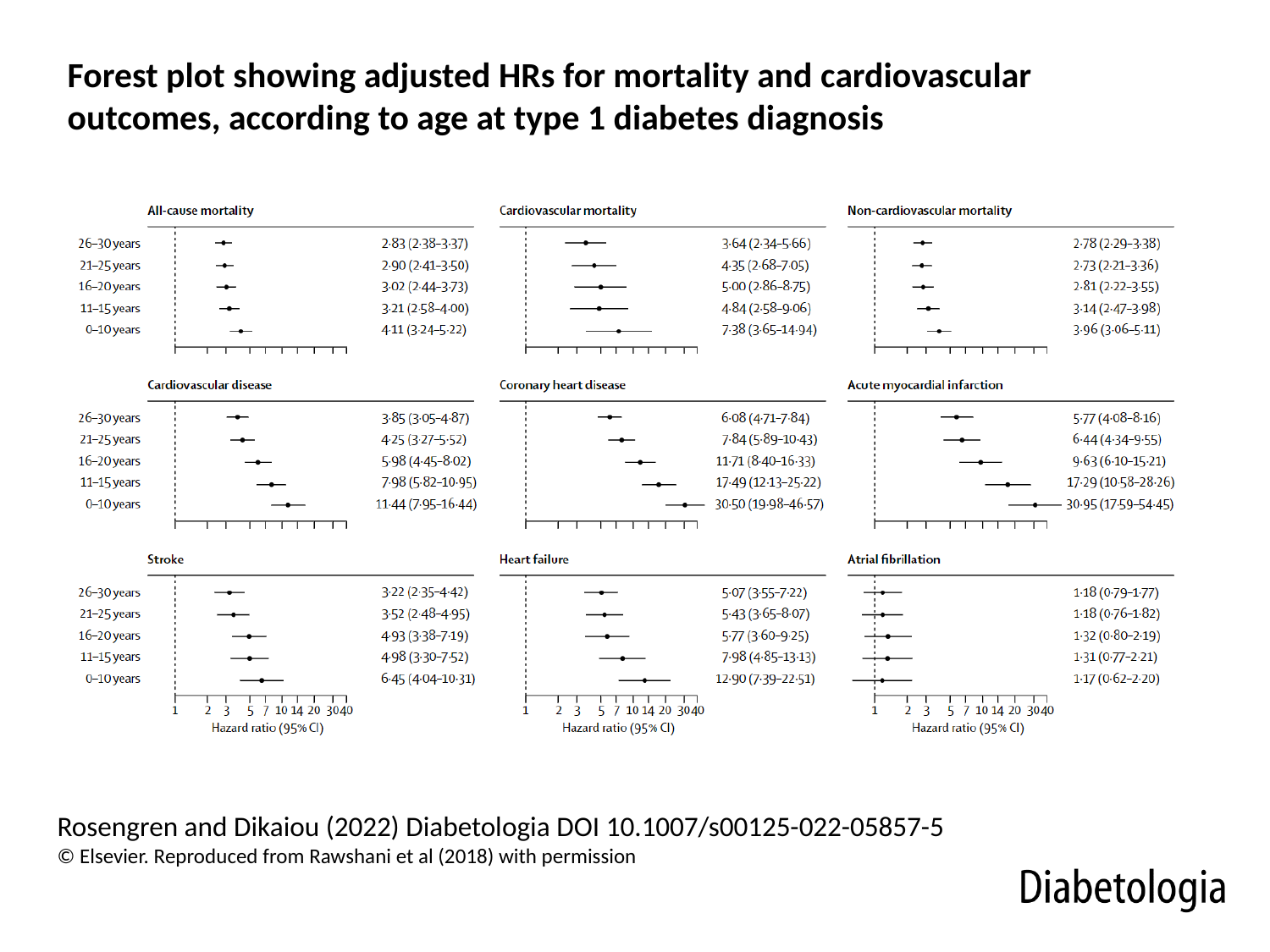

Forest plot showing adjusted HRs for mortality and cardiovascular outcomes, according to age at type 1 diabetes diagnosis
Rosengren and Dikaiou (2022) Diabetologia DOI 10.1007/s00125-022-05857-5
© Elsevier. Reproduced from Rawshani et al (2018) with permission

## Slide 4
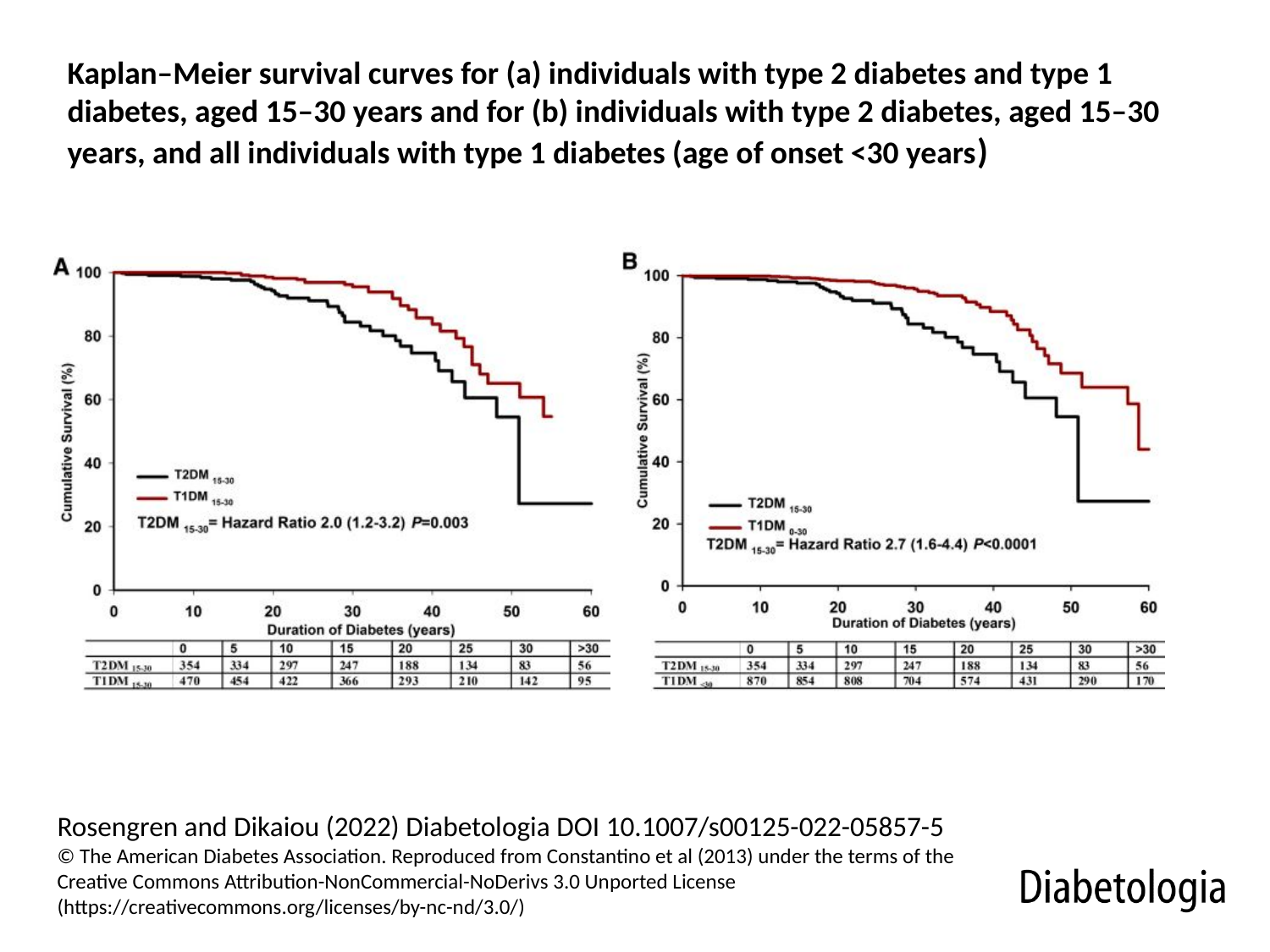

Kaplan–Meier survival curves for (a) individuals with type 2 diabetes and type 1 diabetes, aged 15–30 years and for (b) individuals with type 2 diabetes, aged 15–30 years, and all individuals with type 1 diabetes (age of onset <30 years)
Rosengren and Dikaiou (2022) Diabetologia DOI 10.1007/s00125-022-05857-5
© The American Diabetes Association. Reproduced from Constantino et al (2013) under the terms of the Creative Commons Attribution-NonCommercial-NoDerivs 3.0 Unported License (https://creativecommons.org/licenses/by-nc-nd/3.0/)
